# A Comprehensive Review of Immunosuppression Used for Liver Transplantation

**DOI:** 10.1155/2009/701464

**Published:** 2009-07-16

**Authors:** Sandeep Mukherjee, Urmila Mukherjee

**Affiliations:** ^1^Section of Gastroenterology and Hepatology, University of Nebraska Medical Center, Omaha, NE 68198-3285, USA; ^2^Southampton University Hospital Trust, Tremona Road, Southampton, New Hampshire SO16 6YD, UK

## Abstract

Since liver transplantation was approved for the treatment of end stage liver disease, calcineurin inhibitors (CNI's) have played a critical role in the preservation of allograft function. Unfortunately, these medications cause a variety of Side effects such as diabetes, hypertension and nephrotoxicity which in turn result in significant morbidity and reduced quality of life. A variety of newer immunosuppressants have been evaluated over the last decade in an attempt to either substitute for CNI's or use with reduced dose CNI's while still preserving allograft function However, current data does not recommend complete cessation of CNI's due to unacceptably high rates of allograft rejection. As these medications have their own unique adverse effects, a careful assessment on their risks and benefits is essential, particularly when additive or synergistic effects with CNI's may occur. Furthermore, the impact of these newer medications on the risk of hepatitis C recurrence and progression remains to be elucidated. Controlled trials are urgently required to assist transplant physicians with choosing the optimum immunosuppressive regimen for their patients. This review will discuss commonly used immunosuppressants prescribed in liver transplantation, emerging therapties and where appropriate, the impact of these medications on the recurrence of hepatitis C after liver transplantation.

## 1. Introduction

In the early 1980's, two sentinel events heralded a new era in liver transplantation. The first was the introduction of Cyclosporine (Csa) in 1981 which revolutionized immunosuppression (IS) by drastically reducing the incidence of allograft rejection when combined with corticosteroids (CS) and azathioprine (AZA). This was followed by a pivotal consensus meeting at the National Institutes of Health in 1983 which approved liver transplantation (LT) for the treatment of end stage liver disease [1, 2]. In 1994, a landmark study by the US multicenter FK506 Liver Study Group comparing Csa with tacrolimus reported that although survival with both drugs was similar, tacrolimus was associated with fewer episodes of steroid-resistant rejection at a cost of increased adverse events such as nephrotoxicity and neurotoxicity [3]. Rejection which was reported to be an important cause of death in this study has now become more manageable due to the development of newer and more potent immunosuppressants such that overimmunosuppression has become a greater cause of concern. 

The optimal IS regimen remains the holy grail of organ transplantation until tolerogenic interventions succeed, that is, the level of drug therapy which leads to graft acceptance with least suppression of systemic immunity. This approach is further complicated by a lack of standardization in IS between transplant programs and the management of chronic and, to a lesser extent, acute cellular rejection (ACR) [4]. Current protocols use a combination of drugs with different modes of action and toxicities directed at specific sites of the T-cell activation cascade, thus allowing lower doses of each drug [5]. Induction therapy refers to the practice of administering potent antibody therapy in the perioperative period (when the risk of allograft rejection is greatest) and delaying the introduction of maintenance therapy such as calcineurin inhibitors (CNI's) which have been the backbone of most immunosuppressive regimens in LT. Due to the well-known adverse effects of long-term CNI use, alternative strategies such as CNI minimization or even complete avoidance have been attempted [6–8]. The process of ACR and T cell activation will be briefly reviewed before discussing immunosuppressive drugs used in LT.

## 2. Acute Cellular Rejection

ACR is a complex process comprised of the following steps: alloantigen recognition, T-cell activation, clonal expansion, and graft inflammation.

### 2.1. Allograft Recognition

Foreign (or allo-) antigens are presented to lymphocytes by antigen-presenting cells (APC's) such as dendritic cells. After LT, these antigens are shed into the circulation and presented to secondary lymphoid organs such as the spleen and regional lymph nodes. Naive lymphocytes home to these secondary lymphoid organs via specific receptors and encounter APC's [9, 10]. This process is aborted by antilymphocyte antibodies. APC's enzymatically process foreign proteins and load them onto major histocompatibility complex (MHC) molecules, which are displayed on the cell surface to T cells. The T-cell receptor (TCR) is the antigen-recognition unit on the T-cell surface and associated with molecules such as Cluster of Differentiation 3 (CD3) and either CD4 or CD8 [11]. The TCR-CD3 complex interacts with the peptide fragment carried by the MHC molecule of the APC is stabilized by the CD4 or CD8 molecule and results in Signal 1 of T-cell activation, a calcium-dependent pathway which is unable to activate naive T cells independently.

### 2.2. T-Cell Activation

Signal 2 is a calcium-independent pathway that represents the binding of costimulatory molecules on T cells such as CD28 receptor with their ligands found on APC's which include but are not limited to molecules such as B7.1 (CD80), B7.2 (CD86), and CD40/CD40L (or CD154). Both signals 1 and 2 are required for naive T-cell activation which is primarily mediated by calcineurin, protein kinase C and zeta-associated protein-70 activation of NF-AT, NF-*κ*B, and AP-1, respectively [12]. These cytoplasmic factors translocate to the nucleus and bind to various gene promoters associated with T-cell activation and proliferation, particularly interleukin-2 (IL-2). This initiates the G_0_ to G_1_ transition of the cell cycle which results in T-lymphocyte activation which can be arrested by CNI's such as Csa and tacrolimus.

### 2.3. Clonal Expansion

Signal 3 of T-cell activation results from autocrine and paracrine cytokine-mediated signalling via specific cytokine receptors. The IL-2 receptor family includes receptors for IL-2, IL-4, IL-7, IL-12, and IL-15 and share a common gamma-chain but differ in composition of the alpha and beta chains. In T cells, IL-2 binds to the gamma-chain of the IL-2 receptor and activates the Janus kinases (JAK) 1 and 3. This in turn triggers a cascade of intracellular signalling pathways such as signal transducers and activators of transcription (STAT) 5, Ras-Raf-MAP kinase, and mTOR/P13-K/p70 S6 kinase activation. This results in cell proliferation, deoxyribonucleic acid (DNA) synthesis, and cell division as demonstrated by transition of the cell cycle from G_1_ to the S phase. This important pathway can be interrupted by AZA, mycophenolate mofetil (MMF), sirolimus, and everolimus.

### 2.4. Inflammation

T cell activation and proliferation results in the release of a variety of cytokines which in turn recruit cytotoxic T cells, activated macrophages and B cells, chemokines, and adhesion molecules. A variety of cell adhesion molecules such as CD2, LFA-1, and VLA-4 are also activated while L-selectin is downregulated. The net effect of these processes is to create an inflammatory milieu by developing an environment which attracts activated T cells. Cell damage and death arises from the production of vasoactive and toxic mediators from activated T cells such as tumor necrosis factor-alpha (TNF-alpha), TNF-beta (also known as lymphotoxin), as well as cytotoxins, such as perforin and granzymes, a family of serine proteases and enhanced Fas ligand expression. This process may be inhibited by CS and antilymphocyte antibodie.

## 3. Immunosuppressive Agents

Immunosuppressive medications can be classified in a variety of ways-induction versus maintenance, biologic versus pharmacologic, mechanism of action, and/or site of signal, pathway interruption (Table 1). Biologic agents are often used for induction and include antilymphocyte antibodies (mono- and polyclonal) and anticytokine receptor antibodies. Pharmacologic immunosuppression includes virtually all the other immunosuppressants used for maintenance therapy such as CS, CNI's which act by suppressing cytokine release and cell cycle inhibitors (AZA, MMF, sirolimus, everolimus).

Inhibitors of the Signal 1 pathway include agents that affect T-cell recognition of alloantigen and signal transduction via the calcium-dependent calcineurin pathway. Signal 2 inhibitors inhibit costimulatory pathways, and Signal 3 inhibitors inhibit cytokine-driven proliferation. Finally, other agents inhibit a variety of other points in the immune system such as antimetabolites that interfere with DNA and ribonucleic acid (RNA) replication or lymphocyte trafficking, and investigational agents whose mechanism of action has not fully determined.

## 4. Use of Immunosuppressive Agents in Liver Transplantation in the United States

The Scientific Registry of Transplant Recipients recently reported the nature of the use of IS in the United States by analyzing the United Network of Organ Sharing database [13, 14]. Induction antibody use was noted in 18% of LT, the majority of which were IL-2 receptor antibodies and the remainder being antithymocyte globulins. CNI use was reported in 97% of patients discharged from the hospital after LT in the United States in 2002 while CS use was reported in more than 90% of patients. At discharge, MMF was noted in nearly 48%, AZA in 4% at discharge, and rapamycin in nearly 7% of LT. The popularity of CNI is not only a testament of the effectiveness of these medications in liver transplant recipients but also provides transplant professionals an opportunity to perform well-designed trials using CNI-free IS in an attempt to avoid the long-term effects of CNI toxicity after LTX. Immunosuppressive medications used in LT will now be reviewed and appropriate drug interactions will be discussed.

## 5. Calcineurin Inhibitors—Cyclosporine and Tacrolimus

### 5.1. Background

Cyclosporine (Neoral, Novartis) is a cyclic polypeptide comprised of 11 amino acids (molecular weight 1202.61) and was derived from the fungus *Tolypocladium inflatum * in 1972. Csa's immunosuppressive activity was first discovered in 1976 by Borel et al. who noted an absence of myelotoxicity, a common complication of earlier immunosuppressants [15]. One-year survival following LT was only 26% in 1980 but the introduction of Csa the following year proved to be a breakthrough in IS and led to its approval for use in organ transplantation in 1982 [16–18]. The impact of Csa after LT was confirmed in a retrospective study in which one- and five-year survival was 70 and 63% whereas survival with conventional IS using prednisone and AZA was 33 and 20%, respectively [19].

Tacrolimus (Prograf, FK506, Astellas Pharmaceuticals) is a macrolide compound with a unique hemiketal-masked alpha, beta diketoamide moiety in it structure. It is derived from the fermentation products of fungus *Streptomyces tsukabaensis* and which was discovered by the Fujisawa Pharmaceutical Company in 1984 following analysis of soil samples from Mount Tsukuba in Japan [20]. It was approved for use in LT in the United States in 1994.

### 5.2. Mechanism of Action

Csa and tacrolimus are calcineurin inhibitors that bind to their specific immunophilins, cyclophilin and FK binding protein, respectively, before the drug-receptor complex binds to and inhibits calcineurin, a calcium-dependent phosphatase. Both immunophilins have peptidy-prolyl-cis-trans isomerase activity although this activity is not believed to be related to the immunosuppressive activity of the CNI-immunophilin complex. Csa and tacrolimus are responsible for the dephosphorylation of a variety of transcription factors, particularly nuclear factor of activated T cells (NF-AT), a relatively lymphocyte-specific cyoplasmic-based transcription factor. NF-AT sites are present in the promoter regions of important cytokines such as IL-2, IL-3, IL-4, granulocyte-macrophage colony stimulating factor, interferon gamma (INF-gamma), and TNF-alpha which are critical in the efficient transcription of these cytokine genes in response to activating signals through the T cell receptor. Transforming growth factor beta transcription is also increased with CNI use which may affect the development of fibrosis in transplanted livers. CNI's therefore act primarily by interfering with Signal 2 of T-cell activation. Experimental evidence also suggests that the effectiveness of Csa is due to specific and reversible inhibition of immunocompetent lymphocytes in the G0- or G1-phase of the cell cycle.

### 5.3. Pharmacokinetics and Pharmacodynamics

Csa was originally formulated as Sandimmune, a corn oil-based preparation limited by variable absorption, particularly in the presence of cholestasis. This led to the development and virtually universal adoption of Neoral, a nonaqueous, microemulsified version, by most LT programs. Csa is variably absorbed from the jejunum and enters the lymphatic system. Csa can be administeerd intravenously at a dose approximately 30% of the oral dose. Peak concentrations in blood and plasma are achieved at about 3.5 hours. Csa is distributed largely outside the blood volume with concentrations greatest in adipose, adrenal, hepatic, pancreatic and renal tissues. In blood, Csa distribution is concentration–dependent (33–47% is in plasma, 4–9% in lymphocytes, 5–12% in granulocytes, and 41–58% in erythrocytes) with uptake by erythocytes and leucocytes saturated at higher concentration. In plasma, approximately 90% is bound to proteins, primarily lipoproteins.Csa elimination in blood is biphasic with a half-life of approximately 18 hours (range: 10 to 27 hours). Elimination is primarily biliary with only 6% of the dose excreted in the urine.

Csa is extensively metabolized by the cytochrome P450 3A4 system into metabolites that have virtually no immunosuppressive activity. However, any drug that interacts with the P450 pathway can influence Csa levels. Only 0.1% of the dose is excreted in the urine as unchanged drug. Of 15 metabolites characterized in human urine, only nine have been assigned structures.

Tacrolimus is approximately 100 times more potent than Csa. Oral bioavailability is variable (5–67%) with the rate and extent of absorption greatest under fasting conditions. The presence and composition of food decreases the rate and extent of absorption with the effect most pronounced with a high-fat- meal mean area under the curve (AUC) and peak concentration were reduced by 37% and 77%, respectively. A high-carbohydrate meal decreased mean AUC by 28%. Tacrolimus has a half-life between 31.9 to 48.1 hours and a volume of distribution about 0.85 to 1.94 liters per kilogram (L/kg). Protein binding is about 99%, mainly to albumin and alpha-1 acid glycoprotein. Like Csa, tacrolimus is extensively metabolized by the cytochrome CYP-450 3A4 system with 13-demethyl tacrolimus identified as the major metabolite. Less than 1% of an administered dose is excreted unchanged in the urine.

### 5.4. Side Effects

The main side effects of Csa and tacrolimus are hypertension, nephrotoxicity, neurotoxicity, and lipid abnormalities giving rise to an increased risk of death from cardiovascular complications. Gingival hyperplasia and hirsutism are associated with Csa whereas diabetes is more common with tacrolimus. It has been suggested that the inherent CNI properties of Csa and tacrolimus are linked to nephrotoxicity although recent data suggest inhibition of peptidyl-prolyl-cis trans-isomerase activity may account for nephrotoxicity [21].

### 5.5. Drug Interactions

The following medications include common examples of drug interaction with CNI's and is not a complete list. Examples of some common medications which inhibit the P450 pathway and therefore increase Csa levels include: calcium channel blockers (diltiazem, verapamil, nicardipine); antibiotics (erythomycin, azithromycin, clarithromycin,quinopristin/daltopristin); antifungals (fluconazole, itraconazole, ketoconazole); amiodarone; allopurinol; bromocriptine; colchicines and metoclopramide.

Protease inhibitors used for treating human immunodeficieny virus (e.g., indinavir, nelfinavir, ritonavir, and saquinavir) are known to inhibit cytochrome P-450 3A and thus could potentially increase the concentrations of Csa. However no formal studies of the interaction are available. Care should be exercised when these drugs are administered concomitantly. Grapefruit and grapefruit juice also increase blood concentrations of Csa and should be avoided.

Examples of some common drugs and dietary supplements that decrease CNI levels include: antibiotics (rifampin, nafcillin); anticonvulsants (carbamazepine, phenobarbital, phenytoin); octreotide; orlistat; sulphinpyrazone; terbinafine and ticlodipine. There have been reports of a serious drug interaction between Csa and the herbal dietary supplement, Saint. John's Wort [22]. This interaction has been reported to produce a marked reduction in the blood concentrations of Csa, resulting in subtherapeutic levels, rejection of transplanted organs, and graft loss.

Rifabutin is known to increase the metabolism of other drugs metabolized by the cytochrome P-450 system. The interaction between rifabutin and Csa has not been well studied but care should be exercised when these two drugs are administered concomitantly.

### 5.6. Clinical Use in Liver Transplantation

The incremental improvement in patient and graft survival seen with the introduction of Csa in LT was far more dramatic than the improvement seen with the introduction of tacrolimus [23]. Three prospective, randomized controlled trials have reported a significantly lower incidence of rejection with tacrolimus yet no significant difference in one year patient or graft survival [3, 24, 25]. One criticism of these early studies was their use of oil-based (Sandimmune, Sandoz) as opposed to the microemulsified version (Neoral, Novartis), raising concerns of bioavailability versus efficacy. However, a landmark study by O’Grady et al. appeared to shed some clarity on which CNI might have a greater impact on graft and patient survival [26]. The preliminary one year findings found tacrolimus more beneficial after primary liver transplants in adults with respect to freedom from graft loss and immunological failure. The final data after three years confirmed the significant difference between Csa and tacrolimus although freedom from death or retransplantation no longer achieved statistical significance (relative risk 0.79; 95% Confidence interval CI 0.62–1.02; *P* = .065). A total of 62.1% of patients randomized to tacrolimus were alive at theee years with their original graft and allocated study medication compared to 41.6% in the Csa limb (*P* < .001). A further important finding of the study was the observation no difference was detected between tacrolimus and Csa in hepatitis C- (HCV-) positive patients. These findings were supported in a meta-analysis comparing cyclosporine versus tacrolimus for liver transplant patients [27, 28]. Tacrolimus was superior with respect to patient and graft survival at one year, acute cellular rejection, and steroid resistant rejection. The incidence of lymphoproliferative disease and requirement for dialysis was similar between both groups while diabetes mellitus more common in the tacrolimus group. The investigators also reported more patients stopped Csa than tacrolimus and treating 100 recipients with tacrolimus instead of Csa would prevent rejection and steroid-resistant rejection in nine and seven patients, respectively, and graft loss and death in five and two patients, respectively. However, four additional patients would develop diabetes after LT due to the diabetogenic properties of tacrolimus compared to Csa.

The impact of CNI’S on the natural history of recurrent HCV has gained renewed interest after recent in vitro studies showing an antiviral effect of Csa on HCV replication in the replicon system [29]. This was supported by a retrospective study comparing Csa with tacrolimus in patients who received interferon-based therapy for recurrent HCV [30]. Patients treated with Csa (46%) were more likely to achieve sustained viral response versus patients treated with tacrolimus (27%) (*P* = .03). In addition, not only did Csa inhibit HCV replication in a dose-dependent manner but when combined with interferon had an additive effect independent of interferon signalling. Although there was no statistically significant difference in patient survival between the two groups, Csa-treated patients had a lower baseline HCV RNA and more episodes of acute cellular rejection requiring steroid treatment. However, these findings have not been reproduced in randomized, controlled prospective studies. A recent meta-analysis reported similar rates of fibrosis and patient and graft survival at one year regardless of which calcineurin inhibitor was chosen [31]. The diabetogenic impact of tacrolimus on the natural history of recurrent HCV remains a concern, although a recent study showed no difference in outcomes in Csa versus tacrolimus-treated HCV patients at three years [26]. Currently, a randomized controlled prospective study comparing Csa versus tacrolimus incorporating serial liver biopsies and HCV-RNA levels is underway but until these results are available, it seems reasonable to state that current evidence does not support a beneficial effect of Csa over tacrolimus on HCV recurrence.

## 6. Corticosteroids

### 6.1. Background

Corticosteroids (CS) remain the most widely used non-CNI immunosuppressant in LT. After early pioneering studies showed CS could prolong skin graft survival in rabbits, Starzl et al. and Murray et al. independently demonstrated in 1963 that CS with AZA could extend patient and allograft graft survival after human allograft renal transplantation [32–34].This combination of CS with AZA remained the cornerstone of IS for organ transplantation until the introduction of Csa in the early 1980's. However, CS continue to be used as first line therapy for the treatment of ACR and in patients transplanted for auto immune diseases, often used as an adjunct in maintenance with an agent such as CNI to prevent rejection.

### 6.2. Mechanism of Action

CS act primarily on T cell activation by inhibiting the production of T cell cytokines such as IL-2, IL-6, and interferon-gamma which are required to enhance the response of lymphocytes and macrophages to allograft antigens. They also suppress antibody and complement binding and stimulate the migration of T cells from the intravascular compartment to lymphoid tissue.

### 6.3. Side Effects

A variety of side effects have been reported with corticosteroids (Table 2). Osteoporosis is a common complication with an incidence greatest in the first six months post-LT and patients maintained on more than ten milligrams per kilogram per day should be regularly screened and where appropriate, offered treatment.

### 6.4. Drug Interactions

Most drug interactions with corticosteroids are of little clinical significance. Antacids may reduce absorption of deflazacort (Table 3). Csa increases the plasma concentration of prednisolone while high doses of methylprednisolone increase plasma concentrations of Csa.

### 6.5. Clinical Use in Liver Transplantation

Unfortunately, acute and chronic dosing of corticosteroids are associated with numerous side effects which had led to several attempts to minimize or eliminate their use during the early and late post-LT period. Several attempts to reduce or eliminate CS in corticosteroid use have been undertaken but this requires great care as rapid steroid taper may be associated with an increased incidence of rejection or flare of an underlying disease. Although the use of CS varies between LT programs, they remain an important component of our pharmacological armamentarium although their use as an adjunct in maintenance therapy will be limited to a few cases due to the rapid development of newer and more potent agents which lack their morbidity.

In patients transplanted for HCV, steroids were traditionally withdrawn rapidly (less than three months) and steroid boluses avoided if possible. The concern had been that as the HCV virus had a steroid-responsive element, the use of steroids was associated with enhanced viral replication, and, thus more aggressive viral recurrence. Although this holds true for patients with recurrent HCV who receive steroid boluses, particularly when rejection cannot be unequivocally differentiated from recurrent viral hepatitis, this observation could not be extrapolated to maintenance steroid use. A provocative retrospective study first raised concerns about rapid taper of maintenance steroids in this patient population by reporting more severe recurrent HCV in this subgroup of patients [35]. The investigators analyzed the records of all the patients transplanted for HCV between 1991 and 1997 and followed 80 consecutive patients who survived more than one month after LT for a median of 45 months. 38 patients (47.5%) of patients were diagnosed with recurrent HCV, 22 had severe recurrent disease, and decompensated cirrhosis occurred in six patients (7.5%). The only factor associated with both recurrence and severity of HCV was the method of CS tapering-in patients receiving a higher daily prednisone dose 12 months after transplantation, the proportion of recurrent hepatitis C was 35.7% versus 66.6% (*P* = .02; odds ratio (OR), 3.6; 95% CI: 1.25 to 10.36), and among patients receiving a higher daily prednisone dose, six months after transplantation, the proportion of moderate/severe HCV was 40% versus 89% (*P* = .03; OR: 0.08, 95% CI: 0.008 to 0.84). Prednisone dose at month six was significantly associated with disease-free survival of the liver graft. The same investigators recently reported the results of their prospective randomized study comparing rapid versus slow taper in patients transplanted for HCV and confirmed that rapid tapering was associated with more severe recurrent disease [36]. They recommended that CS should be tapered slowly in these patients using a dose of 2.5–5 mg for up to two years although it remains unclear how long such patients should be treated with steroids [37].

## 7. Rapamycin

### 7.1. Background

Sirolimus (Rapamycin, Wyeth-Ayerst) is a macrolide compound with a molecular weight of 914.2 derived from the actinomycete *Streptomyces hygroscopicus.* It was discovered in soil samples brought from Easter Island (Rapa Nui) by the Canadian Medical Research Expedition between December 1964 and February 1965. Sirolimus has a long history dating from the 1970s at the same time when Csa was discovered and was considered to have novel anti fungal properties. However, it was not further developed as several side effects were noted such as lymphoid tissue regression and immunosuppressive effects in rat models of autoimmune diseases. This led to studies on the efficacy of the compound in mouse and rat organ allograft models in the mid 1980's, particularly after structural homology was noted between sirolimus and tacrolimus. Rapamycin was launched for clinical use in organ transplantation in 1999.

### 7.2. Mechanism of Action

Sirolimus is a potent immunosuppressant which exerts its immunosuppressive effects by inhibiting IL-2 and IL-15 driven proliferation of hematopoietic (B and T cells) and non hematopoietic cells such as vascular smooth muscle cells [38]. Sirolimus also decreases antibody production by B cells. This occurs at relatively low blood levels in vivo. In animal models, sirolimus acts synergistically with Csa suggesting a difference in mechanism of action between sirolimus and calcineurin inhibitors. Despite the structural homology between sirolimus and tacrolimus with both drugs binding to the same intracellular immunophilin, FK506 binding protein, a 12-kDa binding protein (FK Binding protein-12) in T cells, the two drugs act synergistically rather than competitively and also differ in their mechanism of action. Csa and tacrolimus (which inhibit the phosphatase calcineurin after binding to heir respective immunophilins FKBP and cyclophilin, respectively) inhibit early events in T cell activation particularly the expression of IL-2 in the G_0_-G_1_ stage of the cell cycle. Sirolimus which also binds to the FKBP family, particularly FKBP-12, does not bind to calcineurin but instead binds to target molecules with kinase activity called MTOR (mammalian targets of rapamycin), also known as FRAP and RAFT. MTOR plays a key role in the signal transduction pathways downstream to many growth factor receptors (including the IL-2 receptor) and the PI3 kinase/AKT/protein kinase B pathway. TOR and possibly the phosphatase PP2A control the phosphorylation of proteins that regulate the translation of mRNA's encoding regulators of the cell cycle such as translation inhibitor 4E-BP1, eukaryotic translation initiator protein 4G_1_, p70S6kinase, (which in turn phosphorylates 40S ribosomal protein S6) and cyclin-dependent kinases [39]. This may be mediated directly or indirectly as TOR acts partly by regulating PP2A.

The immunosuppressive activity of sirolimus activity is primarily related to blockade of IL-2 and IL-15 induction of B and T cell proliferation via inhibition of p70S6 kinase through high affinity binding to FKBP and prevents progression of the cell cycle from the G_1_ to S phase. In other words, rapamycin allows T cell activation but prevents these cells from proliferating in response to IL-2.This is particularly relevant in lymphoid cell as demonstrated by the observation lymphocyte proliferation is arrested even when rapamycin is administered twelve hours after initiation of stimulation. An interesting effect of sirolimus is its potential anti-tumor effect, presumably by a similar mechanism by which it affects immune cells but in addition there is evidence sirolimus may inhibit angiogenesis. This may occur via inhibition of transcription factors such as hypoxia-inducible factor 1a (H-IF 1a) which results in a decrease in the elaboration of angiogenic molecules such as vascular endothelial growth factor. It may be possible in the future to predict which tumors may be sensitive to mTOR inhibitors by examining the status of the PI3K/AKT pathway of these tumors. However, there is currently insufficient clinical data to support sirolimus use for the prevention and/or treatment of patients with hepato-biliary carcinoma pre or post transplant [40].

Sirolimus has also demonstrated potent anti fungal and anti proliferative properties, independent of immunosuprresive activity. Zhu et al. investigated the effects of sirolimus in a carbon tetrachloride model of hepatic fibrosis in rats and on hepatic stellate proliferation in vitro [41]. The investigators noted that sirolimus inhibited extracellular matrix deposition in a rat model of fibrogenesis as determined by histological analysis, collagen content, messenger RNA levels of procollagen, and transforming growth factor beta-1 and tissue transglutaminase activity. In addition, sirolimus decreased platelet growth factor-induced proliferation of hepatic stellate cells, supporting the hypothesis sirolimus may inhibit hepatic fibrosis and thus prevent or delay the development of cirrhosis. Others have reported that enhanced immuosuppression by sirolimus may actually enhance viral replication but adequately controlled trials evaluating these outcomes have yet to be performed and there is currently insufficient evidence to support or refute either concern. Sirolimus was approved by the U.S. Food and Drug administration only for use in renal transplantation in 1999.

### 7.3. Pharmacokinetics and Pharmacodynamics

A major limitation with sirolimus was the development of a proper oral formulation with acceptable stability, bioavailability and predictability in absorption characteristics. The compound is very lipophilic and hence poorly soluble in water but in oily solution or microemulsion is readily absorbed after oral administration. Sirolimus is also freely soluble in acetone, acetonitrile, benzyl alcohol, and chloroform. Oral absorption is rapid with a bioavailabilty of 14% with oral solution and 18% with tablets. Absorption is reduced in African Americans and in the presence of a high- fat diet. Levy et al. studied the route of administration, effect of bile duct diversion, and time after liver transplantation on 26 liver transplant recipients and reported that none of these factors influenced sirolimus absorption [42]. However, the long half-life of sirolimus and narrow therapeutic window necessitates regular therapeutic drug monitoring (These concerns led to the development of Everolimus, a sirolimus derivative with improved physicochemical properties currently undergoing trials in organ transplant recipients). The 50% inhibitory concentration for sirolimus inhibition of FK506 binding to FK506 binding protein 12 is approximately 0.4–0.9 nano moles per liter (nmol/L).

The volume of distribution of sirolimus is 12 ± 7.52 liters per kilogram of body weight. Extensive uptake occurs in blood cells with up to 95% of uptake occurring in erythrocytes. Sirolimus is extensively bound to plasma proteins (92%), particularly albumin, alpha-one acid glycoprotein, and lipoproteins. Sirolimus undergoes extensive hepatic metabolism by the P450 3A4 cythochrome enzymes and produces the metabolites hydroxysirolimus, demethylsirolimus and hydroxydemethysirolimus. Sirolimus bioavailability and clearance are dependent on intestinal and hepatic metabolism by cytochrome P-450 (CYP) 3A4 enzymes. Elimination occurs in 57 to 63 hours but may be significantly increased up to 72 hours in males although no dosage adjustment is required. Steady state is usually reached five to seven days after dose adjustment. The majority (91%) of the metabolites of sirolimus are eliminated in feces via the multidrug efflux P-glycoprotein pump into the gastrointestinal lumen with only a minor amount (2.2%) recovered from urine. 

Peak blood concentraion in renal transplant recipients was 12.2 ± 6.2 and 37.4 ± 21 nanograms per milliliter (ng/mL) in renal transplant patients administered two mg and five milligrams, respectively, of sirolimus in combination with Csa and CS. Differences in sensitivity and specificity exist between different methods of detection (e.g., immunoassays versus high performance liquid chromatography) with chromatographic methods can yield values 20% lower than whole blood immunoassay levels. Using immunoassays, whole blood concentration averages nine ng/mL in patients who received two mg of sirolimus per day and 17 ng/mL in patients who received five mg of sirolimus per day [43]. Sirolimus pharmacokinetics can also be altered during drug coadministration. For example, when sirolimus is administered concomitantly with the microemulsion formulation of Csa rather than administered separately four hours apart, sirolimus trough levels increase. Furthermore, diltiazem and ketoconazole increase sirolimus peak concentration while rifampin decreases it. Therapeutic drug monitoring is therefore required to achieve the best clinical outcome in selected cases. The immunosuppressant effect of sirolimus can last for up to six months after discontinuation in some animal studies.

### 7.4. Side Effects

The most common side effects are dose- related hyperlipidemia and cytopenias such as thrombocytopenia, anemia and leucopenia. Dyslipedemia has been reported in up to 80% of renal transplant recipients on sirolimus with mean cholesterol levels of 240 mg/dL although the prevalence in liver transplant recipients is reported to be approximately 44% [44]. Trotter et al. reported that hyperlipidemia and hypercholesterolemia were more common and severe in patients on sirolimus-Csa compared to sirolimus-tacrolimus combination therapy [45]. However, it remains unclear if dyslipidemia-associated sirolimus translates into an increased risk of atherosclerosis, a multifaceted, multifactorial complex disease process influenced not only by eleveated levels of serum lipids but underlying inflammation, fibrogenesis and cellular proliferation, processes which may be inhibited by sirolimus.

Unlike CNI's, nephrotoxicty is uncommon although proteinuria leading to nephrotic syndrome has been reported and in renal transplant recipients, sirolimus has been associated with a higher incidence of nephrotoxicity when used with Csa but not tacrolimus [46]. Animal studies have suggested sirolimus enhances the nephrotoxic effects of Csa by increasing renal partitioning of this drug into the renal tubular cell [47]. Arthalgias and aphthous ulcers have also been reported with the use of sirolimus. An uncommon but potentially life-threatening complication of sirolimus is interstitial pneumonia which necessitates immediate cessation and steroids [48]. 

Liver tests abnormalities are occasionally seen and occur a mean of 21 days after initiation of sirolimus [49]. A variety of histological changes have been described including sinusoidal congestion and eosinophilia, all of which reversed within one month of drug discontinuation. Sirolimus has received a black box warning in liver transplantation due to an increased incidence in hepatic artery thrombosis (HAT) in the first post operative month. Wound dehiscence has also been reported in a variety of transplant recipients on sirolimus which has led to substitution of rapamycin for an alternative immunosuppressant in patients undergoing elective surgery and avoidance in the first month of transplantation due to the twin risks of dehiscence and HAT [50]. Despite a single center study in liver transplant recipients showed no evidence of an increased risk of HAT or wound dehiscence with sirolimus, most if not all liver transplant programs avoid sirolimus use in the first post operative month [51, 52].

### 7.5. Drug Interactions

As sirolimus is metabolized by the P450-3A4 microsomal system, drugs which inhibit or induce this system can significantly affect sirolimus metabolism. Common examples of drugs which inhibit sirolimus metabolism leading to potentially toxic levels include erythromycin, fluconazole, and protease inhibitors. Phenytoin has been reported to activate sirolimus metabolism leading to sub therapeutic levels [53].

### 7.6. Clinical Use in Liver Transplantation

Experience with sirolimus in LT is limited in contrast to its use in renal transplantation. It has been used in conjunction with low dose calcineurin inhibitors shortly after transplantation or as monotherapy months or years after transplantation in an attempt to prevent or mitigate calcineurin nephrotoxicity. Watson et al. were the first to report their experience with sirolimus in 15 liver transplant recipients in 1999 [54]. Three different sirolimus-based protocols were used: prednisolone, microemulsion Csa and sirolimus; Csa and sirolimus and sirolimus monotherapy—all patients were on sirolimus monotherapy by three months post-transplant with a follow-up between 117–806 days. Rejection was more common on monotherapy than double therapy but absent on triple therapy. Sirolimus was generally well tolerated with only three patients discontinuing sirolimus: one each for hyperlipidemia, pneumocystis pneumonia, and taste intolerance. The authors concluded sirolimus combined with Csa provided potent immunosuppression of liver allografts with sirolimus monotherapy adequate and well tolerated for maintenance therapy. This seminal work led to an important study by McAlister et al. who reported their experience with sirolimus in combination with tacrolimus in 32 solid organ recipients of whom 23 had undergone LT [55]. Only one patient (3%) experienced ACR who upon further investigation was discovered to have discontinued sirolimus.

Pridohl et al. reported their experience with in 22 patients who received sirolimus, tacrolimus and CS [56] Patient and graft survival at one year were 91% and 78%, respectively. Subsequent studies with larger numbers of patients, albeit non randomized, have been encouraging with excellent patient and survival rates in concert with low rates of acute rejection or steroid-resistant rejection [57, 58]. Trotter et al. compared their experience in 39 patients with Csa or tacrolimus with sirolimus and rapid steroid taper over three days with historical controls [59]. After a mean follow-up of 124 days, they observed a 30% reduction in rate of ACR (48% versus 70%; *P* < .05) and 65% reduction in steroid-resistant rejection (8% versus 37%; *P* < .05).

Several , single-center, non randomized studies have reported improved renal function in liver transplant recipients on CNI's inhibitors switched to either low dose CNI's and sirolimus or sirolimus monotherapy. Fairbanks et al. converted 21 patients on CNI's to sirolimus monotherpay (*n* = 18) and sirolimus with low dose steroids (*n* = 3) and followed patients for a mean of 66.8 ± 38.9 weeks after conversion [60]. Renal function improved in 71% of patients (15 of 21 patients) and median estimated glomerular filtration rate (GFR) improved by 27% (*P* = .01). In a retrospective study of 38 pediatric LT recipients, Casas-Melley et al. reported that patients with renal impairment (11 patients) showed improvement in serum creatinine levels from a mean baseline of 1.3 to 0.8 milligrams per decilitre (mg/dL) with calculated creatinine clearance (Schwartz formula) improving from 63.7 to 84.8 milliliters per minute (mL/min) (*P* = .03) [61] They also reported that patients started on sirolimus for rejection showed significant improvement in hepatocellular enzymes despite a reduction in the tacrolimus level from 12.2 to 7.5 ng/mL. However, this observation was limited by the absence of serial liver biopsies following initiation of sirolimus 

Recent and more elegantly-designed studies have questioned the renal-toxic sparing effects of sirolimus. In a randomized controlled trial of conventional IS with CNI's versus sirolimus-based IS on 30 patients (greater than six months post-liver transplantation), there was a modest improvement in change in GFR following sirolimus conversion at three and twelve months [62]. Although the difference in absolute GFR between the two study arms was significant at three months, this did not hold true at 12 months. These findings were supported by Shenoy et al. who randomized 40 liver transplant recipients with renal dysfunction (24 hour creatinine clearance 40–80 mL/min) to maintenance therapy with CNI's or sirolimus [63]. They noted a significant improvement in creatinine clearance in the sirolimus arm at three months but at 12 month follow -up, there was no statistical difference between the two drugs. Dubay et al. reported the impact of sirolimus on renal dysfunction in an elegantly designed case-control study of LT recipients with renal dysfunction [64]. 57 patients were treated with sirolimus after more than 90 days post operatively and for at least 90 days. The control group consisted of 57 patients on low-dose CNI's inhibitors matched for age, sex and gender. The investigators noted that patients exposed to CNI's for more than five years or those with a creatinine clearance less than 30 mL/min when converted to sirolimus actually fared worse than patients maintained on low dose CNI's. Furthermore, progression to renal replacement therapy, episodes of rejection and death were similar between the two arms although side effects were more common in the sirolimus arm. The authors concluded that there was no advantage to sirolimus conversion in liver transplant recipients with CNI-related nephrotoxicity. These findings suggest the renal-toxic preventing effects of sirolimus are more likely to be achieved when treatment is initiated in the early post-LT period before CNI-nephrotoxicity has developed.

## 8. Purine Synthesis Inhibitors

This section will only review mycophenolic acid as older purine antagonists such as azathioprine and cyclophosphamide are no longer used in LT.

## 9. Mycophenolate Mofetil

### 9.1. Background

Mycophenolate mofetil (MMF) is a morpholinoethyl ester of mycophenolate which is produced by several species of the fungus *Penicillium* and was first discovered in 1893. MMF was developed by Allison and colleagues and is hydrolysed to its active form, mycophenolic acid (MPA) [65]. MMF is available in two forms: MMF (CellCept, Roche) and enteric-coated mycophenolate sodium (Myfortic, Novartis). Although MMF is rapidly absorbed in the stomach before it is converted to MPA, the enteric-coated preparation releases MPA in the small intestine via a pH-dependent mechanism [66]. Interestingly, side effects with the enteric form persist and appear to occur independently of gastrointestinal absorption as confirmed in randomized, double-blind studies [67, 68].

### 9.2. Mechanism of Action

In 1969, MMF was discovered to block *de novo * purine nucleotide synthesis by inhibiting type 2 inosine monophosphate dehydrogenase (IMPDH) and the production of guanosine nucleotides such as guanosine monophosphate (GMP) [69]. MMF inhibits type 2 IMPDH nearly five times more potently than type 1 IMPDH which predominates in resting cells. The mechanism of type two IMPDH inhibition is believed to involve the binding of MMF to the nicotinamide site which prevents the conversion of IMP to GMP [70]. In vitro studies have shown MPA inhibits antibody formation, does not affect production of cytokines implicated in allograft rejection, inhibits transfer of fucose and mannose to glycoproteins including adhesion molecule and inhibits smooth muscle cell proliferation.

Cells depleted of GMP are unable to synthesize guanine triphosphate (GTP) and deoxy guanine triphosphate (dGTP) and therefore cannot replicate unless they are able to maintain GMP levels through the purine salvage pathway. B and T lymphocytes lack a key enzyme of this salvage pathway, hypoxathine guanine phosphoribosyl transferase, and are therefore unable to replicate in the presence of MMF as they cannot bypass this MPA-induced block with cells staying in the S phase of the cell cycle [71]. MMF therefore has a powerful cytostatic effect on lymphocytes by inhibiting both mitogen- and alloantigen-induced stimulation. The effects of MMF can be reversed by guanosine and deoxyguanosine. MMF also reduces recruitment of leucocytes to inflammatory sites.

### 9.3. Pharmacokinetics and Pharmacodynamics

MMF is rapidly absorbed after oral administration and hydrolyzed to MPA. Bioavailability is between 90–94% but food decreases MPA concentrations so MMF should be administered at least one hour before or two hours after meals.

Mean volume of distribution is 3.6l L/kg (intravenous preparation) and 4 L/kg (oral). Protein binding is 97% (MPA) and 82% for MPAG, an inactive metabolite. Free MPA is the pharmacologically active fraction.

MMF is rapidly and completely hydrolysed to MPA which is converted to inactive MPA glucuronide by hepatic glucuronyl transferase. Since genetic variations of this enzyme exist, the effectiveness of MPA and level of glucuronidation may vary. The liver is assumed to be the primary site of MPAG production but it remains unclear if extra-hepatic production occurs. MPAG undergoes enterohepatic recycling and is converted to MPA.

MMF is excreted in MPAG mostly in the urine (93%) and feces (6%). Following oral administration, half-life and plasma clearance of MPA are 17.9 hours and 193 mL/min which is reduced slightly to 16.6 hours and 177 mL/min, respectively following intravenous administration. Liver disease impairs MPA conjugation which prolongs MPA half-life. Interestingly, fluctuations in albumin levels which are common in liver transplant recipients can lead to fluctuations in MMF pharmacokinetics, changes which are not seen commonly in renal transplant recipients [72].

### 9.4. Side Effects

The most common side effects are gastrointestinal (anorexia, abdominal pain, gastritis, diarrhea in up to 30%) and hematological (neutropenia in up to 3%) which are usually dose-related. This requires dose reduction or cessation between 24–57% of patients but if the white cell count does not rise after dose reduction, MMF should be stopped. Although MMF is usually dosed at 1 g twice a day, patients may tolerate the drug better if started at 500 mg twice a day or four times a day. MMF does not cause nephrotoxicity or neurotoxicity.

### 9.5. Drug Interactions

Drugs which may increase plasma concentrations of MPA include acyclovir, ganciclovir, probenecid, salicyaltes and sirolimus. Drugs which may reduce MPA levels include but are not limited to antacids containing aluminium or magnesium, cholestyramine, iron, metronidazole, norfloxacin, and rifampin. MPA also decreases protein binding to phenytoin and theophylline leading to elevated levels of both drugs. MMF also markedly potentiates the anti-herpetic activities of acyclovir and ganciclovir and should not be given with other anti-metabolites such as AZA.

### 9.6. Clinical Use in Liver Transplantation

Although MMF acts similarly to AZA, it is a more effective immunosuppressant with fewer side effects rendering AZA use virtually obsolete in liver transplantation. As MMF does not cause nephrotoxicity or neurotoxicity, it has been widely use as a CNI-sparing agent and has been used in LT since the early 1990's [73–75]. When used in combination with tacrolimus and steroids versus tacrolimus and steroids in a randomized controlled trial, Jain et al. reported that patients in the MMF group experienced fewer episodes of acute cellular rejection, similar patient and graft survival and improved renal function and lesser amount of maintenance steroids [76]. However, the favorable effect of MMF was lost when MMF was stopped which occurred in 37% of patients secondary to infections complications and bone marrow depression. MMF has also been evaluated for the treatment of refractory rejection in liver transplant recipients. In one retrospective study of 47 patients, MMF was added to Csa or tacrolimus with steroid-resistant rejection, of whom 12 did not respond to OKT3 [77]. Liver function tests normalized in 81% of patients (follow-up biopsies were not reported) although gastrointestinal and hematological side effects occurred in 31.4%. Serum creatinine decreased significantly within two weeks of MMF with reduced CNI dosing and creatinine clearance increased within three months in 77.9% of patients treated with MMF and reduced dose CNI. Akamatsu et al. reported that 81% of patients with steroid-resistant rejection after living donor liver transplantation responded to the addition on MMF and only 19% of patients required OKT3 [78]. Although gastrointestinal and hematological side effects were common, there was no increased in the incidence of infections with the authors concluding MMF had a valuable role in the treatment of steroid-resistant rejection before the initiation of monoclonal antibodies.

Data on the impact of azathioprine and MMF on HCV recurrence has been at odds. A well-designed, randomized, prospective study of 106 patients comparing tacrolimus plus steroids vs tacrolimus plus steroids plus MMF showed no effect of MMF on patient or graft survival or HCV recurrence [79]. However, subsequent studies, albeit smaller in size and nonrandomized, have reported worsening HCV RNA viremia with either AZA substitution for MMF or when AZA is compared with MMF [80]. Wiesner et al.  also recently reported that MMF was an important factor in improved outcomes in patients on tacrolimus-based immunosuppression after a multiple regression analysis of 11 670 patients reported to the SRTR [81]. The wide spectrum of reported results is difficult to interpret but nonetheless a common factor in all the earlier-described studies is not whether MMF is superior to AZA but rather the overall intensity of IS may have more of an impact on HCV recurrence than the independent action of either drug.

As MMF is not nephrotoxic, it has a role similar to rapamycin as a calcineurin-sparing agent in patients with renal dysfunction. However, it has no role as monotherapy in liver transplantation due to an unacceptably high incidence of ACR, severe chronic rejection requiring retransplantation and severe steroid-resistant rejection [82, 83].

## 10. Antilymphocytic Antibody Therapy

The original concept of lymphoid depletion in LT was described by Starzl et al. [84]. Subsequent studies demonstrated that antibody therapy to specific antigens on B and T cells will deplete these cell populations although their use has historically been limited to the peri operative period (in an attempt to reduce CNI-mediated nephrotoxicity or acute cellular rejection) and for the treatment of steroid-resistant rejection. Monoclonal and polyclonal antbodies are often classified as “depleting” agents not only for their effect on B and T cells but due to the intravascular release of cytokines from lymphocytes. This can lead to hypotension, fever, brochospasm and tachycardia which can often be treated by pretreatment with steroids, antihistamines, and acetaminophen.

## 11. Monoclonal Anti-T- Cell Receptor Antibodies

### 11.1. Background/Discovery

In 1979, Kung et al. were the first to produce mouse monoclonal antibodies against T cell surface receptor antigens using hybridoma technology [85].

### 11.2. Mechanism of Action

Of the three monoclonal antibodies discovered by Kung et al., one antibody called muromonab-CD3 (OKT3) had defined specificity to the CD3 receptor of the T cell, reacting with more that 95% of peripheral mature T cells without affecting immature thymocytes. Binding of OKT3 to the CD3 complex causes internalization of the CD3 receptor and loss of CD3 positive cells from the periphery which can be monitored by simple laboratory tests-successful OKT3 treatment is associated with a prompt decline in CD3 positive T cells from approximately 60% to less than 5% [86]. Monitoring of the absolute neutrophil count is occasionally used and if levels less than 500 cells per milliliter are not reached, some authorities recommend repeating the OKT3 dose in an attempt to overwhelm the developing antibodies.

### 11.3. Pharmcokinetics and Pharmocodynamics

OKT3 is administered intravenously as a bolus injection and reaches steady-state trough levels by three days. Time to onset of action is within minutes and duration of activity is approximately one week.

### 11.4. Side Effects

Severe side effects are related to the release of proinflammatory cytokines in response to the first few doses of OKT3. They include fever, hypotension, headache from aseptic meningitis, dyspnea from flash pulmonary edema, and gastrointestinal complaints such as nausea, diarrhea, and vomiting. These symptoms may be prevented by pretreatment with antihistamines, acetaminophen and steroids (steroids only used before first dose of OKT3).Onset is usually within one hour and symptoms often resolve within four to six hours. 

Post transplant lymphoproliferative disease (PTLD) may develop more frequently in patients treated with OKT3 and concern has been raised that this complication may occur commonly in patients transplanted for HCV [87]. Two large studies showed an incidence of PTLD of approximately 3% in LT recipients exposed to OKT3-although one of these studies showed a strong association between PTLD and OKT3 exposure, this was not reproducible in the other [88, 89].

### 11.5. Clinical Use

OKT3 was first used in LT in 1987 for treatment of ACR in a multicenter randomized trial [90]. 28 patients who failed to respond to two doses of methylprednisolone boluses were randomized to continue steroid therapy versus switched to OKT3. Three of 13 patients assigned to the steroid group responded promptly and maintained normal liver function for a mean of 9.5 months. OKT3 rescue was required in ten patients who failed to improve despite receiving up to 6 grams of methylprednisolone (mean: 3.3 grams per patient). One patient died of sepsis and hepatic failure. Rejection was reversed in nine OKT3-rescue patients, seven of whom are well 1–17 months later. In the OKT3 group, improved allograft function was observed within 72 hours in 11 of 15 patients. Two patients with inadequate response were successfully rescued with steroids; one patient underwent retransplantation; and one patient developed a biliary fistula that eventually resulted in sepsis and death. Overall, 82% of patients were alive with the original allograft 1–17 (mean 7.8) months after treatment for acute rejection with one patient alive 14 months following retransplantation. Eighteen (78%) of the survivors required OKT3 as initial [11] or rescue (seven) therapy, whereas only five were successfully managed with steroids. This early trial demonstrated that OKT3 was superior to steroids for reversing liver allograft rejection and may reduce the need for retransplantation even in recipients selected on the basis of having failed initial steroid therapy.

A subsequent multicenter randomized trial was performed to compare the incidence of ACR and renal failure between two immunosuppressive protocols [91]. 46 patients were randomized to a 14-day treatment with OKT3 in association with CS and AZA, Csa being progressively introduced on day 11 post-LT. Fifty patients were randomized to a standard protocol of Csa with CS and AZA. Minimum follow-up was one year and graft and patient survivals were updated for the purpose of the study. The cumulative one-year incidence of ACR was greater in the Csa group (75%) than in the OKT3 group (67%), especially when patients who did not receive full-course treatment with OKT3 were excluded (59%). Renal function was better preserved during the first two postoperative weeks in the OKT3 group than in the control group but plasma creatinine levels were comparable in both groups thereafter. Surprisingly, incidence of severe infections was lower in the OKT3 group (13.6%) than in the Csa group (32%) although the four year incidences of patient and graft survival in the OKT3 group (69% and 61%, resp.) were not different from those in the Csa group (62% versus 54%, resp.). This prospective trial showed OKT3 immunoprophylaxis may be a reasonable alternative to Csa immunoprophylaxis in unselected recipients of a first liver graft.

In addition to the cytokine-mediated side effects of OKT3, a serious side effect includes rapid acceleration of HCV replication leading to severe recurrent disease. In a retrospective study from 1997, Rosen analyzed outcomes for two groups of patients with steroid resistant rejection, of whom one group received OKT3 (*n* = 19) and the other was OKT3 naïve (*n* = 33) [92]. Both groups were matched for age, gender, immunosuppression, and HCV status. The investigators observed that not only did HCV recur earlier in the OKT3-treated group but disease severity was significantly greater with a higher proportion of patients developing cirrhosis. This landmark study has significantly altered the use of OKT3 in liver transplant recipients with recurrent HCV—at present, polyclonal antibodies are used as an alternative to monoclonal antibodies if the diagnosis of acute rejection is unequivocal and has not responded to other standard interventions.

## 12. Polyclonal Antibodies

### 12.1. Background

Although anti lymphocyte antibodies have been in clinical use for the last 2 decades, their use in liver transplantation, particularly with polyclonal anti bodies, continues to evolve. Polyclonal antilymphocyte antibody preparations are heterologous preparations. Animals (including rabbits and horses) are immunized with human T cells and thymocytes, and antisera are collected. A purified gamma globulin fraction (antithymocyte globulin) is used to reduce the likelihood of serum sickness. The US Food and Drug Administration approved antithymocyte globulin preparations are ATGAM of equine origin (Pfizer, New York) and thymoglobulin of rabbit origin (Genzyme, Boston). The limitations of the antithymocyte globulin preparations are related to variability in potency; reactivity to formed blood elements leading to leukopenia, thrombocytopenia, or anemia; and serum sickness. Only antithymocyte globulin of rabbit origin (ATG) will be discussed as the use of ATGAM in the United States for LT is not common.

### 12.2. Mechanism of Action

ATG is a rabbit-derived polyclonal antibody directed against human thymocytes which leads to depletion of peripheral lymphocytes and peripheral lymphopenia. Characterization of the antigen specificity of these preparations suggests that multiple cell surface molecules are recognized by the polyclonal antibody preparations, and the varying specificities may account for the variations in their clinical efficacy [93]. The antibodies are directed not only to multiple T cell antigens such as CD2, CD3, CD4, CD8, CD28, and the T cell receptor but also to antigens on B cells (CD20 and CD40), HLA classes 1and 2, macrophages and natural killer (NK) cells [94]. These polyclonal antibodies operate via antibody-mediated opsonisation and cytolysis, cell depletion by apoptosis, complement mediated cell lysis and internalization of the cell surface receptors. The biological effect of the depleting antibodies are profound and can last longer than heterologous antibodies [95].

There is also evidence from in vitro in and vivo studies that ATG thereapy results in expansion of regulatory T cells [96–98]. This may allow any remaining lymphocytes and/or new lymphocytes to engage the transplanted liver and either recognize the donor organ as self or develop an adaptive immune response which prevents hepatic allograft rejection. Clinical studies are underway to test this hypothesis and to determine whether ATG may promote allograft tolerance.

### 12.3. Pharmacokinetics and Pharmacodynamics

ATG has a half life of approximately three days.

### 12.4. Side Effects

ATG is tolerated better than OKT3 although premedication with anti histamines and acetaminophen is commonly performed Leucopenia and thrombocytopenia are common side effects which are dose-related although serum sickness has also been reported.

### 12.5. Clinical Use

ATG has been used as an induction agent and steroid-sparing agent in LT and also used to treat steroid-resistant rejection. An early randomized controlled trial by Eason et al. compared outcomes of induction with ATG (*n* = 36) versus CS (*n* = 35) followed by maintenance IS with tacrolimus and MMF, with or without steroids, in both groups [99]. Overall survival and graft rates were 91% and 89% for both groups. 20.5% of patients treated with ATG had biopsy-proven rejection successfully treated with increasing tacrolimus doses. 32% of patients administered steroid induction had biopsy-proven rejection of whom 64% required additional steroids for treatment whereas 36% were successfully treated by increasing tacrolimus doses. The incidence of rejection was not statistically significant although there was a significant difference in the incidence of steroid-requiring rejection (*P* = .01). A further important finding of this study was that the incidence of recurrent HCV was 50% in ATG patients versus 71% in steroid patients although this was not statistically significant. Overall incidence and severity of infectious complications were slightly lower in ATG patients due to a greater incidence of cytomegalovirus (CMV) infection in the steroid patients. The findings from this prospective randomized trial supported the use of ATG as a reasonable alternative induction agent to steroid boluses. 

The same investigators reported a two year follow -up of patients from this study which also included 48 additional patients [100]. This study, however, was complicated by new modifications in the IS regimen. In patients who received steroid induction, steroids were tapered over three months. MMF was discontinued over three months in the first 71 patients and over two weeks in the next cohort of 48 patients, leaving tacrolimus monotherapy as the default immunosuppressant as early as two weeks post-LT. Endpoints of the study were survival, rejection, infectious complications, post-LT diabetes, and recurrent HCV. One year patient survival was 85% in both groups while one year graft survival was 82% in the ATG group and 80% in the steroid group (*P* = not significant). Although the incidence of rejection was not significantly different, 50% of patients in the steroid group required pulse steroids to reverse the rejection compared with only one patient (1.6%) in the ATG group (*P* = .03). The incidence of cytomegalovirus infection (*P* < .05) and post-LT diabetes was higher in the steroid group (*P* = .03) with a trend toward decreased severity of hepatitis C virus in the ATG group.

To evaluate the impact of ATG on recurrence of HCV, Nair et al. randomized 64 patients to induction therapy with steroids versus ATG followed by maintenance tacrolimus and compared liver function tests, HCV RNA levels and histology at three-six months post-LT [101]. All patients received antiviral therapy with interferon alpha 2b and ribavirin in the absence of clear contraindications. There was no statistically significant difference between the two groups in terms of inflammation at three months, peak ALT or HCV RNA. The survival between the two groups of patients was also similar suggesting that steroid-free liver transplantation with ATG induction has no negative influence on HCV recurrence after a short-term follow-up. These findings were echoed by De Ruvo et al. following a retrospective analysis aimed at discovering any relationship between RATG immunosuppression and the pattern of recurrence of HCV [102]. The investigators compared outcomes between 22 HCV patients who received ATG induction plus tacrolimus monotherapy with 30 HCV patients receiving transplants within the same year who received conventional tacrolimus plus CS. Patient survival and acute rejection rate did not differ between the two groups. Significantly lower dosages and levels of tacrolimus were possible with ATG and a progressive weaning of tacrolimus monotherapy was accomplished in most patients, without major rejection complications. The HCV recurrence rate was similar in both groups, although significantly lower HCV RNA loads were obtained with ATG pretreatment. The mean time to histological recurrence was shorter in ATG-treated patients but no significant difference was observed in mean Ishak's histologic grading and staging of HCV recurrence. 

These studies continue to reinforce the importance of ATG as a steroid-sparing agent during the induction phase of LT and for the treatment of ACR. Furthermore, ATG does not appear to have a significant impact on HCV recurrence and is also much better tolerated than monoclonal antibodies such as OKT3. These properties of ATG, together with an absence nephrotoxicity, had endeared many LT programs to adopt its use-clinical trials in LT are currently underway to study if ATG induction plus tacrolimus, MMF and CS versus standard of care plus tacrolimus, MMF and CS will impact of renal function and HCV recurrence.

## 13. Alemtuzumab/Campath-1H

Alemtuzumab or campath-1H (C-1H) is a humanized, recombinant anti-CD52 monoclonal antibody. It targets antigen CD52, a cell surface glycoprotein, expressed on more than 95% of peripheral blood lymphocytes (T cells greater than B cells), monocytes, macrophages, and natural killer cells [103]. Alemtuzumab produces profound depletion of circulating lymphocytes but spares stem cells this may last approximately one month and lymphocyte recovery, especially CD4 cells, is slow [104]. A further distinguishing feature of alemtuzumab is its ability to deplete lymphocytes from both the blood and lymph nodes with the exception of plasma and memory cells [105]. The rationale for its initial use was to facilitate lower doses of maintenance immunosuppression while higher doses of alemtuzumab were subsequently used to induce tolerance and eliminate maintenance immunosuppression [106]. Although alemtuzumab alone caused profound lymphocyte and monocyte depletion, it did not prevent the development of ACR [107].

The University of Miami reported their experience with alemtuzumab induction combined with low-dose tacrolimus alone in LT recipients [108]. This group was compared with patients receiving standard doses of tacrolimus and CS. Alemtuzumab induction resulted in significantly improved renal function compared with the control subjects. Although the incidence of ACR was significantly lower with alemtuzumab induction in the first two months (15% in the study group versus 46% in the control group) after LT, the median time to rejection was longer with the majority of ACR episodes occurring after two months. This was most likely related to the time it took lymphocyte counts to recover from alemtuzumab. The overall incidence of ACR was not significantly different between the two groups (46% in the alemtuzumab group vs. 55% in control subjects) although alemtuzumab-related side effects were minimal. Further studies have focused on its use in combination with other low-dose immunosuppressive therapy but only in the presence of CNI's as alemtuzumab monotherapy was associated with a high incidence of vascular rejection [109].

## 14. Interleukin-2 Receptor Antibodies

### 14.1. Mechanism of Action

Two IL-2 receptor (IL-2R) monoclonal antibodies in clinical use are basiliximab (Simulect, Novartis) and daclizumab (Zenapax, Hoffman-La Roche)) which are chimeric Ig G1 monoclonal antibodies (daclizumab is 90% human and 10% murine) produced by recombinant DNA technology. Their chimeric structure renders these agents less immunogenic than other monoclonal antibodies such as OKT3. The IL-2 receptor is composed of three noncovalently bound chains: CD25 (a 55 kilodalton kD alpha chain); a 75 kD beta chain and a 64 kD gamma chain. Both agents engage the alpha chain of the IL-2 receptor (also referred to as CD25 or T cell activation antigen) which is upregulated in activated T lymphocytes, resulting in internalization of the T cell receptor. As CD25 is not involved in transmembrane signaling, antibodies to the alpha chain will not result in agonist effects [110]. Although IL-2 is still able to bind to its receptor, this affinity is greatly reduced leading to inhibition of T cell proliferation in response to circulating IL-2 (or inhibition of Signal 3 of the T cell activation pathway).

### 14.2. Pharmacokinetics and Pharmacodynamics

The half-life of basiliximab is 7.2 days with a volume of distribution in adults 8.6 ± 4.1 liters and clearance of 41 mL/min. Daclizumab has an estimated half-life of 20 days and a volume of distribution between 0.032 and 0.043 liters/kilogram. Half-life and volume of distribution are decreased in LT recipients compared to renal transplant recipients due to a higher volume of distribution in patients with ascites and increased immunoglobulin clearance from hypersplenism [111]. The receptor suppressive effects of basiliximab persist for 3–4 weeks while those of daclizumumab last for up to 10 weeks.

### 14.3. Clinical Use

The role of IL-2R monoclonal antibodies in LT continues to evolve. Early studies used these agents primarily to reduce or avoid the use of CNI's (particularly in patients with renal dysfunction) or to eliminate corticosteroid use entirely. Early trials comparing basiliximab combined with Csa, AZA, and CS or used with Csa and steroids were associated with an ACR rate of 35% [112, 113]. It was suggested this high rate was related to activated T cells bypassing CD25 blockade as immunohisochecmical staining demonstrated the presence of activated T cells infiltrating the hepatic allograft [114]. To evaluate the impact of basiliximab on HCV recurrence in which the primary end point was histological recurrence of HCV at 12 months, Filliponi et al. randomized 140 patients to basiliximab and steroids versus basiliximab and placebo followed by Csa and AZA in both groups [115]. Histological recurrence (defined as an Ishak score greater than 12) was 41.2% with basiliximab and steroids versus 37.5% with basiliximab and placebo (*P* = .354) together with a lower treatment failure rate in the steroid group. A prospective study with long-term follow-up by Schmeding et al. revaluated the incidence of ACR, graft and patient survival in patients treated with basiliximab plus CNI's and steroids (*n* = 51) versus CNI's and steroids (*n* = 48) [116]. The investigators did not detect a difference in these primary end points between the two groups except for a modest improvement in renal function in the basiliximab group.

Several studies have reported that the combination of daclizumab induction with delayed low-dose TAC is safe and effective in patients with renal dysfunction [117, 118]. However, current data suggests daclizumab should not be used without CNI's. Hirose et al. reported ACR of 100% in CNI naive patients when comparing daclizumab, MMF and steroids versus daclizumab, MMF and delayed CNI [119]. 57.1% of these patients developed steroid-resistant rejection requiring OKT3. Possible explanations for the high incidence of rejection, which remain to be proven, included increased elimination in ascites which may have contributed to the short half-life compared to renal transplant recipients in which the half-life is usually between three and four weeks.

To evaluate the impact of daclizumab on fibrosis from HCV recurrence at 1 year, Kato el at. randomized patients to tacrolimus and daclizumab (steroid-free) versus tacrolimus and CS during 1999–2001 and then tacrolimus, MMF and daclizumab (steroid-free) versus. tacrolimus, MMF and CS during 2002–2005 [120]. No noticeable differences in mean fibrosis stage between the two treatment arms. The incidence of ACR during the first year was the only factor associated with a significantly increased fibrosis stage at 1 year (*P* = 0.0003); stage two or greater was seen in 63% (17 of 27) versus. 19% (8 of 43) of those with versus to those without rejection. In addition, MMF use was associated with significantly fewer patients experiencing ACR during the first six and 12 months post-LT (*P* = .006 and .046, resp.). Post-LT diabetes mellitus occurred in 10% versus 45%, and wound infection in 6% versus 31% of steroid-free versus CS patients (*P* = 0.003 and 0.01, respectively). The authors concluded that although patients tolerated the steroid-free protocol with fewer side effects, its use had no apparent impact on hepatic fibrosis progression. The occurrence of ACR rejection was strongly associated with increased hepatic fibrosis at one year. 

Klintmalm et al. recently reported results of a two-year prospective randomized study evaluating the impact of IS on recurrent HCV progression and incidence of rejection [121]. Recurrent HCV was defined as the presence of at least grade three inflammation or at least stage twp fibrosis in liver biopsies performed within the first year according to the classification described by Batts and Ludwig. The investigators randomized 312 patients to three arms: arm one (*n* = 80): tacrolimus plus prednisone; arm two (*n* = 79): tacrolimus plus prednisone plus MMF; arm three (*n* = 153); daclizumab plus tacrolimus plus MMF. Protocol biopsies were performed at 90, 365, and 720 days. At the two year follow-up, there was no statistical difference in the incidence of rejection, HCV RNA viremia, HCV recurrence, patient survival, or graft survival among the three groups. However, more accelerated HCV recurrence was observed between years one and two in arms one and two. The investigators concluded that although HCV recurrence at two years was not influenced by IS, the rate of progression in year two may have been influenced by steroids and MMF and recommended longer-term follow-up evaluation for these patients.

Until adequately powered randomized controlled trials are performed, the use of IL-receptor antibodies in LT should be used with caution and under the rigor of a clinical trial, Although these agents are generally welltolerated, they are expensive, have yet to show a clear benefit over standard treatment and not recommended for routine use for induction therapy.

## 15. Immunosuppressants in Development

CNI's remain the most widely used medications for maintenance therapy. However, due to their well-known side effects, a variety of alternatives have been considered in an attempt to either minimize or possibly abolish its use entirely. Most of the studies that have evaluated these newer immunosuppressives have been conducted in renal transplant recipients—only when promising results are obtained are such trials repeated in LT recipients. Nonetheless, it is important for health care professionals responsible for the care of LT patients to be at least aware of some of the latest and most promising drugs being studied today in the event they may provide us with a CNI-free era in the future (Table 4).

## 16. FK 778

FK778 (Astellas, Osaka, Japan) is a synthetic malononitrilamide derived from an active metabolite of leflunomide, an isoxazole derivative with anti-inflammatory, immunosuppressive and antiproliferative properties [122, 123]. It binds to dihydro-orotate dehydrogenase and inhibits de novo pyrimidine biosynthesis, thus blocking B- and T-cell proliferation and also strongly suppresses IgG and IgM antibody production [124] It possesses antiviral properties and can inhibit cytomegalovirus and polyoma virus replication in vitro [125]. A vasculoprotective effect with inhibition of neointima formation, thought to be associated with inhibition of tyrosine kinase and platelet derived growth factor stimulated smooth muscle proliferation, has also been reported in animal studies [126]. Pharmacokinetic studies have shown almost 100% bioavailability with rapid absorption after oral administration. FK778 is metabolized mainly in the liver and only a fraction of drug is excreted unchanged in the urine.

Phase 1 (United States) and phase 2 (Europe) renal transplantation trials have shown that administration with tacrolimus and CS is associated with a reduced rate of ACR [12]. Anemia is the most common side effect which is reversible with dose reduction and/or discontinuation. Studies on LT patients are currently being conducted in Europe.

## 17. JAK3 Inhibitors

Janus (named after the two-faced God of Gates, Janus) activated kinases (JAK's) are vital intermediaries between cytokine receptors and signal transcription and activation of transduction (STAT), which result in activation of the immune cells [127]. JAK-1 and JAK-2 are activated by a broad range of cytokines that use gp130 whereas JAK-3 which is highly expressed in lymphoid cells is activated only by cytokines that bind to gamma-chain-containing receptors. These cytokines include IL-2, IL-4, IL-7, IL-9, IL-15, and IL-21. Stimulation of JAK-3, a cytoplasmic tyrosine kinase, leads to dimerization of STAT 5 transcription factor which is specific for the IL-2 family of cytokines and hence make this a potential target for immunosuppressive drugs [128]. The potential for IS by JAK3 inhibitors was discovered serendipitously by the observation gamma-chain mutations of JAK3 were associated with severe combined immunodeficiency in humans, a condition associated with non functional B cells and an absence of T cells and natural killer (NK) cells. 

However, several concerns exist with JAK3 inhibition. Firstly, loss of NK cells and down regulation of IL-15 transmission may lad to impaired innate immunity and memory. JAK3 is also homologous with JAK2 which mediates signalling by hematopoietic cytokines potentially leading to anemia, leucopenia, and thrombocytopenia [129]. CP-690,550, a JAK3 inhibitor, is currently undergoing phase 2 trials in renal transplant recipients versus tacrolimus with all patients also being maintained on MMF and steroids. It is anticipated encouraging results from these studies will lead to randomized studies in LT recipients. A number of JAK inhibitors, with varying degrees of specificity for JAK-3, are under investigation [130, 131]. Specific JAK-3 inhibitors, which have demonstrated immunosuppressive activity in small animal models, exhibit fewer of the hematological side effects seen with those agents that also inhibit JAK-2.

## 18. FTY 720

FTY 720 represents a new class of immunosuppressive agent that alters cellular homing patterns. It is a synthetic analogue of sphingosine which is isolated from the ascomycete *Isaria sinclairi. * It shares structural and functional homology with sphingosine-1-phosphate (SIP), a natural ligand of several G-protein coupled receptors. It decreases the number of circulating blood lymphocytes with lymphocyte counts reach less than 30% of baseline value within 72 hours of treatment. FTY 720 monophosphate (FTY 720-P), the active form of the drug, acts as a high affinity agonist at the G-protein-coupled SIP receptor family, particularly SIP-1 and SIP-5, on lymphocytes and thymocytes inducing aberrant receptor internalization. This renders the cells unresponsive to the serum lipid SIP-1 receptor and although these cells remain functional, they are sequestered to the lymphoid compartment and unable to migrate to inflammatory tissues such as graft sites [132]. There are also studies that suggest that FTY 720 may provide a level of protection from ischemia/reperfusion injury in livers [133].

Its major side effect identified in phase one and two trials was a negative chronotropic effect, possibly a direct effect of the drug on the sinus node, and exacerbated in the presence of beta-blockade. FTY 720 failed phase three clinical trials and is no longer in clinical development in transplantation. Despite these concerns, this pathway merits attention as it remains a potential target for immunosuppressive drugs.

## 19. Belatacept-Costimulatory Signal Blockade

Signal 2 is a costimulatory signal in T cell activation representing ligation of a receptor on a T cell with its ligand on an antigen-presenting cell. Failure to activate this co-stimulatory pathway leads to T cells becoming immunologically unresponsive—they are unable to replicate, secrete inflammatory cytokines and undergo apoptosis. One of the best studied co stimulatory pathways is the interaction between the T cell receptor CD28 with CD80 (B 7-1) and CD86 (B 7-2) on the APC. The homologue to CD28 is CTLA4 which is transiently expressed after T cell activation and negatively regulates T cell activation. CTLA4 competetively inhibits binding of CD28 to CD80 and 86.

Trials of CTLA4Ig, a soluble recombinant immunoglobulin fusion protein composed of the extracellular fragment of CTLA4 and the Fc portion of immunoglobulin G_1_, were abandoned due to disappointing results in non human primate models. This led to the development of belatacept (LEA29Y), a modified version of CTLA4-Ig with greater affinity to CD80 and CD86 that is conveniently administered monthly [134]. Belatacept consists of a soluble recombinant immumoglobulin fusion protein comprised of the extracellular CTLA-4 domain and the Fc portion of immunoglobulin G_1_.binds to CD80 and CD86 at least four times more potently than its parent compound and has demonstrated at least ten time more potent inhibition of T cell activation in vitro. A recent study compared belatacept versus Csa in combination with CS, MMF and basiliximab in 218 renal transplant recipients [135]. At six months, there was no significant difference in the incidence of ACR between both groups but renal function and chronic allograft nephropathy were significantly better in patients in the belatacept arm. Hypertension and metabolic complications such as hyperlipidemia and post-transplant hyperglycemia were also less common in the belatacept arm. Concerns that concomitant administration of CNI or steroids antagonize the tolerogenic effect of costimulatory blockade remain theoretical at present and hopefully future studies in LT will put these concerns to rest.

## 20. Summary

Rapid advances in the development of immunosuppressive medications promises that the future of LT will continue to be bright. However, in parallel with these discoveries are an urgent need to perform well-designed randomized studies and also to consider the emerging role of pharmacogenomics which has quickly played an important role in the use of drugs such a AZA. It is likely that recurrent HCV will continue to play a pivotal role in LT for the foreseeable future and transplant professionals will need to be aware of the impact future drugs may have on this disease.

## Figures and Tables

**Table 1:** Commonly used immunosuppressive agents in liver transplantation and their target pathways.

Immunosuppressive agent	Target pathway
Pharmacological	

Corticosteroids	(a) Inhibits cytokine transcription by antigen presenting cell
	(b) Selective lysis of immature cortical thymocytes
Calcineurin inhibitors (cyclosporine/neoral and tacrolimus/Prograf/Fk506)	Inhibits Signal 2 transduction via T cell receptor
Mammalian Target of rapamycin inhibitors (sirolimus/rapamycin, everolimus)	Inhibits signal 3 transduction via IL-2 receptor
Azathioprine (Imuran)	Inhibits purine and DNA synthesis
Mycophenolic acid (cell cept)	Inhibits purine and DNA synthesis

Biological	

Anti-CD3 monoclonal antibodies (OKT3)	(a) Causes depletion and receptor modulation in T cell
	(b) Interferes with signal 1
Antithymocyte globulin (ATG)	(a) Causes depletion and receptor modulation in T cells
	(b) Interferes with signal 1, 2 and 3
	(c) Inhibits lymphocyte trafficking
Anti IL-2 alpha chain receptor antibodies (Basiliximab, Daclizumumab)	Inhibits T cell proliferation to IL-2 (signal 3)
Anti-CD52 monoclonal antibodies (campath 1-H)	Causes depletion of thymocytes, T cells, B cells (not plasma cells) and monocytes

**Table 2:** Side effects of corticosteroids.

Cardiovascular	Sodium and fluid retention, hypertension
Gastroenterological	Pancreatitis with high dose steroids, peptic ulcer
Neurologic	Psychosis, altered mood states, headaches, pseudotumor
Opthalmic	Posterior subcapsular cataracts, increased intraocular pressure, glaucoma, exopthalmos
Musculoskeletal	Osteoporosis, vertebral and femoral fractures, aseptic necrosis of femoral head. Myopathy, muscle weakness
Endocrine	Diabetes mellitus/glucose intolerance, Cushingoid facies, hyperlipidemia, growth retardation, menstrual irregularities, suppression of pituitary-adrenal axis
Skin	Acne, increased bruising, impaired wound healing
Infectious	Increased risk of infections, including fungal

**Table 3:** Equivalent doses of steroids.

Glucocorticoid	Dose (milligrams)
Hydrocortisone	20
Deflazacort	6
Prednisolone	5
Prednisone	5
Methylprednisolone	4
Triamcinolone	4
Dexamethasone	0.75

**Table 4:** Investigational immunosuppressive agents in liver transplantation.

Immunosuppressive agent	Target pathway
FK778	Interferes with pyrimidine metabolism and DNA synthesis
JAK inhibitors	Interfere with Signal 3 transduction
FTY720	Inhibits T cell migration to venule endothelial cells in secondary lymphoid tissue
LEA29Y	Interferes with Signal 2 via inhibition of B7/CD28 interaction

## References

[1] (1983). Liver transplantation. National Institutes of Health Consensus Development. *National Institutes of Health Consensus Development Conference Summary*.

[2] Calne RY, White DJ, Thiru S (1985). Cyclosporin G: immunosuppressive effect in dogs with renal allografts. *The Lancet*.

[3] The U.S. Multicenter FK506 Liver Study Group (1994). A comparison of tacrolimus (FK 506) and cyclosporine for immunosuppression in liver transplantation. *The New England Journal of Medicine*.

[4] Demetris AJ (2006). Liver biopsy interpretation for causes of late liver allograft dysfunction. *Hepatology*.

[5] Sharma VK, Li B, Khanna A, Sehajpal PK, Suthanthiran M (1994). Which way for drug-mediated immunosuppression?. *Current Opinion in Immunology*.

[6] Gonwa TA, Mai ML, Melton LB (2001). End-stage renal disease (ESRD) after orthotopic liver transplantation (OLTX) using calcineurin-based immunotherapy: risk of development and treatment. *Transplantation*.

[7] Ojo AO, Held PJ, Port FK (2003). Chronic renal failure after transplantation of a nonrenal organ. *The New England Journal of Medicine*.

[8] Jain AB, Yee LD, Nalesnik MA (1998). Comparative incidence of *de novo* nonlymphoid malignancies after liver transplantation under tacrolimus using surveillance epidemiologic end result data. *Transplantation*.

[9] Zheng H, Jin B, Henrickson SE, Perelson AS, Von Andrian UH, Chakraborty AK (2008). How antigen quantity and quality determine T-cell decisions in lymphoid tissue. *Molecular and Cellular Biology*.

[10] Rubin B, Riond J, Leghait J, Gairin JE (2006). Interactions between CD8*α β* and the TCR*α β*/CD3-receptor complex. *Scandinavian Journal of Immunology*.

[11] Choudhuri K, Wiseman D, Brown MH, Gould K, van der Merwe PA (2005). T-cell receptor triggering is critically dependent on the dimensions of its peptide-MHC ligand. *Nature*.

[12] O'Shea JJ, Johnston JA, Kehrl J, Koretzky G, Samelson LE (2001). Key molecules involved in receptor-mediated lymphocyte activation. *Current Protocols in Immunology*.

[13] Organ Procurement and Transplantation Network/Scientific Registry of Transplant Recipients Annual Report: Transplant Data 1993–2002. http://www.ustransplant.org/cgi-bin/ar?p=data_tables_10.htm&y=2003.

[14] Kaufman DB, Shapiro R, Lucey MR, Cherikh WS, Bustami RT, Dyke DB (2004). Immunosuppression: practice and trends. *American Journal of Transplantation*.

[15] Borel JF, Feurer C, Gubler HU, Stahelin H (1976). Biological effects of cyclosporin A: a new antilymphocytic agent. *Agents and Actions*.

[16] Starzl TE, Koep L, Porter KA (1980). Decline in survival after liver transplantation. *Archives of Surgery*.

[17] Starzl TE, Klintmalm GBG, Porter KA, Iwatsuki S, Schroter GP (1981). Liver transplantation with use of cyclosporin A and prednisone. *The New England Journal of Medicine*.

[18] Calne RY (1981). Liver transplantation. *Annals of Clinical Research*.

[19] Gordon RD, Shaw BW, Iwatsuki S (1986). Indications for liver transplantation in the cyclosporine era. *Surgical Clinics of North America*.

[20] Goto T, Kino T, Hatanaka H (1987). Discovery of FK-506, a novel immunosuppressant isolated from Streptomyces tsukubaensis. *Transplantation Proceedings*.

[21] Hong F, Lee J, Piao YJ (2004). Transgenic mice overexpressing cyclophilin A are resistant to cyclosporin A-induced nephrotoxicity via peptidyl-prolyl cis-trans isomerase activity. *Biochemical and Biophysical Research Communications*.

[22] Murakami Y, Tanaka T, Murakami H, Tsujimoto M, Ohtani H, Sawada Y (2006). Pharmacokinetic modelling of the interaction between St John's wort and ciclosporin A. *British Journal of Clinical Pharmacology*.

[23] Todo S, Fung JJ, Starzl TE (1994). Single-center experience with primary orthotopic liver transplantation with FK 506 immunosuppression. *Annals of Surgery*.

[24] Fung JJ, Eliasziw M, Todo S (1996). The Pittsburgh randomized trial of tacrolimus compared to cyclosporine for hepatic transplantation. *Journal of the American College of Surgeons*.

[25] Williams R (1994). Randomised trial comparing tacrolimus (FK506) and cyclosporin in prevention of liver allograft rejection. *The Lancet*.

[26] O'Grady JG, Hardy P, Burroughs AK (2007). Randomized controlled trial of tacrolimus versus microemulsified cyclosporin (TMC) in liver transplantation: poststudy surveillance to 3 years. *American Journal of Transplantation*.

[27] McAlister VC, Haddad E, Renouf E, Malthaner RA, Kjaer MS, Gluud LL (2006). Cyclosporin versus tacrolimus as primary immunosuppressant after liver transplantation: a meta-analysis. *American Journal of Transplantation*.

[28] Haddad EM, McAlister VC, Renouf E, Malthaner R, Kjaer MS, Gluud LL (2006). Cyclosporin versus tacrolimus for liver transplanted patients. *Cochrane Database of Systematic Reviews*.

[29] Henry SD, Metselaar HJ, Lonsdale RCB (2006). Mycophenolic acid inhibits hepatitis C virus replication and acts in synergy with cyclosporin A and interferon-*α*. *Gastroenterology*.

[30] Firpi RJ, Zhu H, Morelli G (2006). Cyclosporine suppresses hepatitis C virus in vitro and increases the chance of a sustained virological response after liver transplantation. *Liver Transplantation*.

[31] Berenguer M, Royuela A, Zamora J (2007). Immunosuppression with calcineurin inhibitors with respect to the outcome of HCV recurrence after liver transplantation: results of a meta-analysis. *Liver Transplantation*.

[32] Morgan JA (1951). The influence of cortisone on the survival of homografts of skin in the rabbit. *Surgery*.

[33] Starzl TE, Marchioro TL, Waddell WR (1963). The reversal of rejection in human renal homografts with subsequent development of homograft tolerance. *Surgery Gynecology & Obstetrics*.

[34] Murray JE, Merrill JP, Harrison JH, Wilson RE, Dammin GJ (1963). Prolonged survival of human-kidney homografts by immunosuppressive drug therapy. *The New England Journal of Medicine*.

[35] Brillanti S, Vivarelli M, De Ruvo N (2002). Slowly tapering off steroids protects the graft against hepatitis C recurrence after liver transplantation. *Liver Transplantation*.

[36] Vivarelli M, Burra P, Barba GL (2007). Influence of steroids on HCV recurrence after liver transplantation: a prospective study. *Journal of Hepatology*.

[37] Brown RS (2007). Steroids in recurrent hepatitis C following liver transplantation: pitfall or panacea?. *Journal of Hepatology*.

[38] Neuhaus P, Klupp J, Langrehr JM (2001). mTOR inhibitors: an overview. *Liver Transplantation*.

[39] Raught B, Gingras A-C, Gygi SP (2000). Serum-stimulated, rapamycin-sensitive phosphorylation sites in the eukaryotic: translation initiation factor 4GI. *The EMBO Journal*.

[40] Zimmerman MA, Trotter JF, Wachs M (2008). Sirolimus-based immunosuppression following liver transplantation for hepatocellular carcinoma. *Liver Transplantation*.

[41] Zhu J, Wu J, Frizell E (1999). Rapamycin inhibits hepatic stellate cell proliferation in vitro and limits fibrogenesis in an in vivo model of liver fibrosis. *Gastroenterology*.

[42] Levy GA, Grant D, Paradis K, Campestrini J, Smith T, Kovarik JM (2001). Pharmacokinetics and tolerability of 40-0-[2-hydroxyethyl]rapamycin in de novo liver transplant recipients. *Transplantation*.

[43] Holt DW, Lee T, Jones K, Johnston A (2000). Validation of an assay for routine monitoring of sirolimus using HPLC with mass spectrometric detection. *Clinical Chemistry*.

[44] Groth CG, Backman L, Morales J-M (1999). Sirolimus (rapamycin)-based therapy in human renal transplantation: similar efficacy and different toxicity compared with cyclosporine. *Transplantation*.

[45] Trotter JF, Wachs ME, Trouillot TE (2001). Dyslipidemia during sirolimus therapy in liver transplant recipients occurs with concomitant cyclosporine but not tacrolimus. *Liver Transplantation*.

[46] Kahan BD (2000). Efficacy of sirolimus compared with azathioprine for reduction of acute renal allograft rejection: a randomised multicentre study. *The Lancet*.

[47] Napoli KL, Wang M-E, Stepkowski SM, Kahan BD (1998). Relative tissue distributions of cyclosporine and sirolimus after concomitant peroral administration to the RAT: evidence for pharmacokinetic interactions. *Therapeutic Drug Monitoring*.

[48] Flores-Franco RA, Luevano-Flores E, Gaston-Ramirez C (2007). Sirolimus-associated desquamative interstitial pneumonia. *Respiration*.

[49] Neff GW, Ruiz P, Madariaga JR (2004). Sirolimus-associated hepatotoxicity in liver transplantation. *Annals of Pharmacotherapy*.

[50] Dean PG, Lund WJ, Larson TS (2004). Wound-healing complication after kidney transplantation: a prospective, randomized comparison of sirolimus and tacrolimus. *Transplantation*.

[51] Dunkelberg JC, Trotter JF, Wachs M (2003). Sirolimus as primary immunosuppression in liver transplantation is not associated with hepatic artery or wound complications. *Liver Transplantation*.

[52] Fung J, Marcos A (2003). Rapamycin: friend, foe, or misunderstood?. *Liver Transplantation*.

[53] Fridell JA, Jain AKB, Patel K (2003). Phenytoin decreases the blood concentrations of sirolimus in a liver transplant recipient: a case report. *Therapeutic Drug Monitoring*.

[54] Watson CJE, Friend PJ, Jamieson NV (1999). Sirolimus: a potent new immunosuppressant for liver transplantation. *Transplantation*.

[55] McAlister VC, Gao Z, Peltekian K, Domingues J, Mahalati K, MacDonald AS (2000). Sirolimus-tacrolimus combination immunosuppression. *The Lancet*.

[56] Pridohl O, Heinemann K, Hartwig T (2001). Low-dose immunosuppression with FK 506 and sirolimus after liver transplantation: 1-year results. *Transplantation Proceedings*.

[57] McAlister VC, Peltekian KM, Malatjalian DA (2001). Orthotopic liver transplantation using low-dose tacrolimus and sirolimus. *Liver Transplantation*.

[58] MacDonald AS (2003). Rapamycin in combination with cyclosporine or tacrolimus in liver, pancreas, and kidney transplantation. *Transplantation Proceedings*.

[59] Trotter JF, Wachs M, Bak T (2001). Liver transplantation using sirolimus and minimal corticosteroids (3-day taper). *Liver Transplantation*.

[60] Fairbanks KD, Eustance JA, Fine D, Thuluvath PJ (2003). Renal function improves in liver transplant recipients when switched from a calcineurin inhibitor to sirolimus. *Liver Transplantation*.

[61] Casas-Melley AT, Falkenstein KP, Flynn LM, Ziegler VL, Dunn SP (2004). Improvement in renal function and rejection control in pediatric liver transplant recipients with the introduction of sirolimus. *Pediatric Transplantation*.

[62] Watson CJE, Gimson AES, Alexander GJ (2007). A randomized controlled trial of late conversion from calcineurin inhibitor (CNI)-based to sirolimus-based immunosuppression in liver transplant recipients with impaired renal function. *Liver Transplantation*.

[63] Shenoy S, Hardinger KL, Crippin J (2007). Sirolimus conversion in liver transplant recipients with renal dysfunction: a prospective, randomized, single-center trial. *Transplantation*.

[64] Dubay D, Smith RJ, Qiu KG, Levy GA, Lilly L, Therapondos G (2008). Sirolimus in liver transplant recipients with renal dysfunction offers no advantage over low-dose calcineurin inhibitor regimens. *Liver Transplantation*.

[65] Platz KP, Sollinger HW, Hullett DA, Eckhoff DE, Eugui EM, Allison AC (1991). RS-61443—a new, potent immunosuppressive agent. *Transplantation*.

[66] Pescovitz MD, Conti D, Dunn J (2000). Intravenous mycophenolate mofetil: safety, tolerability, and pharmacokinetics. *Clinical Transplantation*.

[67] Budde K, Curtis J, Knoll G (2004). Enteric-coated mycophenolate sodium can be safely administered in maintenance renal transplant patients: results of a 1-year study. *American Journal of Transplantation*.

[68] Schmidt H, Brockmann J (2007). MMF to EC-MPS conversion: what makes the difference. *Liver Transplantation*.

[69] Franklin TJ, Cook JM (1969). The inhibition of nucleic acid synthesis by mycophenolic acid. *Biochemical Journal*.

[70] Sintchak MD, Fleming MA, Futer O (1996). Structure and mechanism of inosine monophosphate dehydrogenase in complex with the immunosuppressant mycophenolic acid. *Cell*.

[71] Hood KA, Zarembski DG (1997). Mycophenolate mofetil: a unique immunosuppressive agent. *American Journal of Health-System Pharmacy*.

[72] Jain A, Venkataramanan R, Hamad IS (2001). Pharmacokinetics of mycophenolic acid after mycophenolate mofetil administration in liver transplant patients treated with tacrolimus. *Journal of Clinical Pharmacology*.

[73] Stegall MD, Wachs ME, Everson G (1997). Prednisone withdrawal 14 days after liver transplantation with mycophenolate: a prospective trial of cyclosporine and tacrolimus. *Transplantation*.

[74] Klupp J, Bechstein WO, Platz KP (1997). Mycophenolate mofetil added to immunosuppression after liver transplantation—first results. *Transplant International*.

[75] Barkmann A, Nashan B, Schmidt HHJ (2000). Improvement of acute and chronic renal dysfunction in liver transplant patients after substitution of calcineurin inhibitors by mycophenolate mofetil. *Transplantation*.

[76] Jain AB, Hamad I, Rakela J (1998). A prospective randomized trial of tacrolimus and prednisone versus tacrolimus, prednisone, and mycophenolate mofetil in primary adult liver transplant recipients: an interim report. *Transplantation*.

[77] Pfitzmann R, Klupp J, Langrehr JM (2003). Mycophenolatemofetil for immunosuppression after liver transplantation: a follow-up study of 191 patients. *Transplantation*.

[78] Akamatsu N, Sugawara Y, Tamura S, Matsui Y, Kaneko J, Makuuchi M (2006). Efficacy of mycofenolate mofetil for steroid-resistant acute rejection after living donor liver transplantation. *World Journal of Gastroenterology*.

[79] Jain A, Kashyap R, Demetris AJ, Eghstesad B, Pokharna R, Fung JJ (2002). A prospective randomized trial of mycophenolate mofetil in liver transplant recipients with hepatitis C. *Liver Transplantation*.

[80] Zekry A, Gleeson M, Guney S, McCaughan GW (2004). A prospective cross-over study comparing the effect of mycophenolate versus azathioprine on allograft function and viral load in liver transplant recipients with recurrent chronic HCV infection. *Liver Transplantation*.

[81] Wiesner RH, Shorr JS, Steffen BJ, Chu AH, Gordon RD, Lake JR (2005). Mycophenolate mofetil combination therapy improves long-term outcomes after liver transplantation in patients with and without hepatitis C. *Liver Transplantation*.

[82] Schlitt HJ, Barkmann A, Boker KHW (2001). Replacement of calcineurin inhibitors with mycophenolate mofetil in liver-transplant patients with renal dysfunction: a randomised controlled study. *The Lancet*.

[83] Stewart SF, Hudson M, Talbot D, Manas D, Day CP (2001). Mycophenolate mofetil monotherapy in liver transplantation. *The Lancet*.

[84] Starzi TE, Koep LJ, Halgrimson CG (1979). Liver transplantation—1978. *Transplantation Proceedings*.

[85] Kung PC, Goldstein G, Reinherz EL, Schlossman SF (1979). Monoclonal antibodies defining distinctive human T cell surface antigens. *Science*.

[86] Chinnakotla S, Klintmalm G, Bussutil R, Klintmalm G (2005). Induction and maintenance of immunosuppression. *Transplantation of the Liver*.

[87] McLaughlin K, Wajstaub S, Marotta P (2000). Increased risk for posttransplant lymphoproliferative disease in recipients of liver transplants with hepatitis C. *Liver Transplantation*.

[88] Sanchez EQ, Marubashi S, Jung G (2002). De novo tumors after liver transplantation: a single-institution experience. *Liver Transplantation*.

[89] Kremers WK, Devarbhavi HC, Wiesner RH, Krom RAF, Macon WR, Habermann TM (2006). Post-transplant lymphoproliferative disorders following liver transplantation: incidence, risk factors and survival. *American Journal of Transplantation*.

[90] Cosimi AB, Cho SI, Delmonico FL, Kaplan NM, Rohrer RJ, Jenkins RL (1987). A randomized clinical trial comparing OKT3 and steroids for treatment of hepatic allograft rejection. *Transplantation*.

[91] Farges O, Ericzon B-G, Bresson-Hadni S (1994). A randomized trial of OKT3-based versus cyclosporine-based immunoprophylaxis after liver transplantation. Long-term results of a European and Australian multicenter study. *Transplantation*.

[92] Rosen HR, Shackleton CR, Higa L (1997). Use of OKT3 is associated with early and severe recurrence of hepatitis C after liver transplantation. *American Journal of Gastroenterology*.

[93] Rebellato LM, Gross U, Verbanac KM, Thomas JM (1994). A comprehensive definition of the major antibody specificities in polyclonal rabbit antithymocyte globulin. *Transplantation*.

[94] Michallet M-C, Preville X, Flacher M, Fournel S, Genestier L, Revillard J-P (2003). Functional antibodies to leukocyte adhesion molecules in antithymocyte globulins. *Transplantation*.

[95] Mueller TM (2003). Thymoglobulin: an immunologic overview. *Current Opinion in Organ Transplantation*.

[96] Feng X, Kajigaya S, Solomou EE (2008). Rabbit ATG but not horse ATG promotes expansion of functional CD4^+^CD25^+^FOXP3^+^ regulatory T cells in vitro. *Blood*.

[97] Lopez M, Clarkson MR, Albin M, Sayegh MH, Najafian N (2006). A novel mechanism of action for anti-thymocyte globulin: induction of CD4^+^CD25^+^Foxp3^+^ regulatory T cells. *Journal of the American Society of Nephrology*.

[98] Lytton SD, Denton CP, Nutzenberger AM (2007). Treatment of autoimmune disease with rabbit anti-T lymphocyte globulin: clinical efficacy and potential mechanisms of action. *Annals of the New York Academy of Sciences*.

[99] Eason JD, Loss GE, Blazek J, Nair S, Mason AL (2001). Steroid-free liver transplantation using rabbit antithymocyte globulin induction: results of a prospective randomized trial. *Liver Transplantation*.

[100] Eason JD, Nair S, Cohen AJ, Blazek JL, Loss GE (2003). Steroid-free liver transplantation using rabbit antithymocyte globulin and early tacrolimus monotherapy. *Transplantation*.

[101] Nair S, Loss GE, Cohen AJ, Eason JD (2006). Induction with rabbit antithymocyte globulin versus induction with corticosteroids in liver transplantation: impact on recurrent hepatitis C virus infection. *Transplantation*.

[102] De Ruvo N, Cucchetti A, Lauro A (2005). Preliminary results of a “prope” tolerogenic regimen with thymoglobulin pretreatment and hepatitis C virus recurrence in liver transplantation. *Transplantation*.

[103] Frampton JE, Wagstaff AJ (2003). Alemtuzumab. *Drugs*.

[104] Calne RY (2000). Prope tolerance: a step in the search for tolerance in the clinic. *World Journal of Surgery*.

[105] Magliocca JF, Knechtle SJ (2006). The evolving role of alemtuzumab (Campath-1H) for immunosuppressive therapy in organ transplantation. *Transplant International*.

[106] Calne R, Friend P, Moffatt S (1998). Prope tolerance, perioperative campath 1H, and low-dose cyclosporin monotherapy in renal allograft recipients. *The Lancet*.

[107] Kirk AD, Hale DA, Mannon RB (2003). Results from a human renal allograft tolerance trial evaluating the humanized CD52-specific monoclonal antibody alemtuzumab (Campath-1H). *Transplantation*.

[108] Tzakis AG, Tryphonopoulos P, Kato T (2004). Preliminary experience with alemtuzumab (Campath-1H) and low-dose tacrolimus immunosuppression in adult liver transplantation. *Transplantation*.

[109] Knechtle SJ, Pirsch JD, Fechner JH (2003). Campath-1H induction plus rapamycin monotherapy for renal transplantation: results of a pilot study. *American Journal of Transplantation*.

[110] Vincenti F (1999). Targeting the interleukin-2 receptor in clinical renal transplantation. *Graft*.

[111] Moser MAJ (2002). Options for induction immunosuppression in liver transplant recipients. *Drugs*.

[112] Calmus Y, Scheele JR, Gonzalez-Pinto I (2002). Immunoprophylaxis with basiliximab, a chimeric anti-interleukin-2 receptor monoclonal antibody, in combination with azathioprine-containing triple therapy in liver transplant recipients. *Liver Transplantation*.

[113] Neuhaus P, Clavien P-A, Kittur D (2002). Improved treatment response with basiliximab immunoprophylaxis after liver transplantation: results from a double-blind randomized placebo-controlled trial. *Liver Transplantation*.

[114] Kwekkeboom J, Warle MC, Rietveld J, Segeren KCA, Tilanus HW, Metselaar HJ (2003). Blockade of intragraft IL-2 receptor-*α* by basiliximab is insufficient to prevent activation of liver graft infiltrating cells. *Transplant Immunology*.

[115] Filipponi F, Callea F, Salizzoni M (2004). Double-blind comparison of hepatitis C histological recurrence rate in HCV+ liver transplant recipients given basiliximab + steroids or basiliximab + placebo, in addition to cyclosporine and azathioprine. *Transplantation*.

[116] Schmeding M, Sauer IM, Kiessling A (2007). Influence of basiliximab induction therapy on long term outcome after liver transplantation, a prospectively randomised trial. *Annals of Transplantation*.

[117] Emre S, Gondolesi G, Polat K (2001). Use of daclizumab as initial immunosuppression in liver transplant recipients with impaired renal function. *Liver Transplantation*.

[118] Eckhoff DE, McGuire B, Sellers M (2000). The safety and efficacy of a two-dose daclizumab (Zenapax) induction therapy in liver transplant recipients. *Transplantation*.

[119] Hirose R, Roberts JP, Quan D (2000). Experience with daclizumab in liver transplantation: renal transplant dosing without calcineurin inhibitors is insufficient to prevent acute rejection in liver transplantation. *Transplantation*.

[120] Kato T, Gaynor JJ, Yoshida H (2007). Randomized trial of steroid-free induction versus corticosteroid maintenance among orthotopic liver transplant recipients with hepatitis C virus: impact on hepatic fibrosis progression at one year. *Transplantation*.

[121] Klintmalm G, Fasola CG, Jennings L (2007). Hepatitis C-3 study: does immunosuppression affect the progression of fibrosis of HCV recurrence after liver transplantation. *Hepatology*.

[122] Birsan T, Dambrin C, Klupp J, Stalder M, Fitzimmons WE, Morris RE (2003). Effects of the malononitrilamide FK778 on immune functions in vitro in whole blood from non-human primates and healthy human volunteers. *Transplant Immunology*.

[123] Bilolo KK, Ouyang J, Wang X (2003). Synergistic effects of malononitrilamides (FK778, FK779) with tacrolimus (FK506) in prevention of acute heart and kidney allograft rejection and reversal of ongoing heart allograft rejection in the rat. *Transplantation*.

[124] Jin MB, Nakayama M, Ogata T (2002). A novel leflunomide derivative, FK778, for immunosuppression after kidney transplantation in dogs. *Surgery*.

[125] Knight DA, Hejmanowski AQ, Dierksheide JE, Williams JW, Chong AS-F, Waldman WJ (2001). Inhibition of herpes simplex virus type 1 by the experimental immunosuppressive agent leflunomide. *Transplantation*.

[126] Savikko J, von Willebrand E, Hayry P (2003). Leflunomide analogue FK778 is vasculoprotective independent of its immunosuppressive effect: potential applications for restenosis and chronic rejection. *Transplantation*.

[127] O'Shea JJ (1997). Jaks, STATs cytokine signal transduction and immunoregulation: are we there yet?. *Immunity*.

[128] Ozaki K, Spolski R, Feng CG (2002). A critical role for IL-21 in regulating immunoglobulin production. *Science*.

[129] Borie DC, Changelian PS, Larson MJ (2005). Immunosuppression by the JAK3 inhibitor CP-690,550 delays rejection and significantly prolongs kidney allograft survival in nonhuman primates. *Transplantation*.

[130] Changelian PS, Flanagan ME, Ball DJ (2003). Prevention of organ allograft rejection by a specific Janus kinase 3 inhibitor. *Science*.

[131] Saemann MD, Zeyda M, Diakos C (2003). Suppression of early T-cell-receptor-triggered cellular activation by the Janus kinase 3 inhibitor WHI-P-154. *Transplantation*.

[132] Brinkmann V, Cyster JG, Hla T (2004). FTY720: sphingosine 1-phosphate receptor-1 in the control of lymphocyte egress and endothelial barrier function. *American Journal of Transplantation*.

[133] Anselmo DM, Amersi FF, Shen X-D (2002). FTY720 pretreatment reduces warm hepatic ischemia reperfusion injury through inhibition of T-lymphocyte infiltration. *American Journal of Transplantation*.

[134] Larsen CP, Pearson TC, Adams AB (2005). Rational development of LEA29Y (belatacept), a high-affinity variant of CTLA4-Ig with potent immunosuppressive properties. *American Journal of Transplantation*.

[135] Vincenti F, Larsen C, Durrbach A (2005). Costimulation blockade with belatacept in renal transplantation. *The New England Journal of Medicine*.

